# Imaging Sites of Inhibition of Proteolysis in Pathomimetic Human Breast Cancer Cultures by Light-Activated Ruthenium Compound

**DOI:** 10.1371/journal.pone.0142527

**Published:** 2015-11-12

**Authors:** Suelem D. Ramalho, Rajgopal Sharma, Jessica K. White, Neha Aggarwal, Anita Chalasani, Mansoureh Sameni, Kamiar Moin, Paulo C. Vieira, Claudia Turro, Jeremy J. Kodanko, Bonnie F. Sloane

**Affiliations:** 1 Department of Chemistry, Federal University of São Carlos, São Carlos, São Paulo, Brazil; 2 Department of Chemistry, Wayne State University, Detroit, Michigan, United States of America; 3 Department of Chemistry and Biochemistry, The Ohio State University, Columbus, Ohio, United States of America; 4 Department of Physiology, School of Medicine, Wayne State University, Detroit, Michigan, United States of America; 5 Department of Pharmacology, School of Medicine, Wayne State University, Detroit, Michigan, United States of America; 6 Department of Oncology, School of Medicine, Wayne State University, Detroit, Michigan, United States of America; INRS, CANADA

## Abstract

The cysteine protease cathepsin B has been causally linked to progression and metastasis of breast cancers. We demonstrate inhibition by a dipeptidyl nitrile inhibitor (compound **1)** of cathepsin B activity and also of pericellular degradation of dye-quenched collagen IV by living breast cancer cells. To image, localize and quantify collagen IV degradation in real-time we used 3D pathomimetic breast cancer models designed to mimic the *in vivo* microenvironment of breast cancers. We further report the synthesis and characterization of a caged version of compound **1**, [Ru(bpy)_2_(**1**)_2_](BF_4_)_2_ (compound **2**), which can be photoactivated with visible light. Upon light activation, compound **2**, like compound **1**, inhibited cathepsin B activity and pericellular collagen IV degradation by the 3D pathomimetic models of living breast cancer cells, without causing toxicity. We suggest that caged inhibitor **2** is a prototype for cathepsin B inhibitors that can control both the site and timing of inhibition in cancer.

## Introduction

Cancer is one of the foremost causes of death worldwide [1]. Breast cancer is the most prevalent type of cancer in women and the leading cause of cancer death in both developed and developing countries. Breast cancer is not a single disease but consists of several subtypes. Triple negative breast cancer (TNBC), a subtype that does not express estrogen receptor (ER) or progesterone receptor (PR) and in which human epidermal growth factor receptor 2 (HER2) is not amplified, is very aggressive, usually affecting young women and representing 15–20% of all cases of breast cancer. At present there are no targeted therapies for TNBC [2–4] so there is an unmet need for new therapeutic strategies.

The tumor microenvironment has a major role in modulating the metastatic capacity of most cancers [5]. Nonetheless the *in vivo* characteristics of the tumor microenvironment are not represented in studies using purified enzymes or cancer cells cultured in two-dimensional (2D) monolayers. In contrast, three-dimensional (3D) cell cultures take into consideration interactions of cells with the extracellular matrix (ECM), cell polarity and cell-to-cell contacts, providing a more accurate context in which to evaluate compound activity and protease inhibition [6–8]. Studies using two distinct approaches (2D and 3D cell culture models) demonstrate the value of evaluating compounds in 3D cell culture models as results in 3D are more comparable to results obtained in *in vivo* models [8].

Cysteine cathepsins are a family of 11 human cysteine proteases that are highly expressed in a variety of cancers [9–12], including breast cancer [13]. Besides being mainly found intracellularly in lysosomes, some cysteine cathepsins are secreted and bind to the surface of cancer cells [9,12,14]. One of these is cathepsin B (CTSB), which plays a key role in facilitating tumor progression, growth, invasion and metastasis [9–13,15]. Targeting proteases such as CTSB that are causal in cancer with conventional small molecule protease inhibitors will be challenging because cysteine cathepsins are crucial housekeeping enzymes that are required for normal cell function throughout the body.

Compounds that release biologically active agents upon irradiation with light can be used to garner spatial and temporal control over biological activity [16–18]. This method, also known as photocaging, is essential for basic research applications *in vitro* and *in vivo* [19]. Photocaging also shows great potential in photochemotherapy, where pharmacologically active compounds are released only in a desired location, reducing the risk of side effects in surrounding tissues [20]. Photocaging groups based on transition metals are attractive for photochemotherapy applications [21–22] because they can be released with visible light [23], as opposed to organic protecting groups that usually require UV light for cleavage [18]. Of the various classes of metal-based protecting groups, Ru^II^(bpy)_2_ has been used widely, due to its excellent visible light absorption and photoreactivity, to allow for release of neurotransmitters [24–28] and cytotoxic agents [29] as well as nitrile-based cysteine protease inhibitors [30–31].

In this study we report inhibition of CTSB by a dipeptidyl nitrile-based inhibitor caged by complexation to the Ru^II^(bpy)_2_ fragment. We used a photoactivation strategy and several methods to confirm inhibitory activity such as activity assays of purified CTSB and human TNBC cell lysates and a live-cell proteolysis assay of TNBC cell lines grown in 3D MAME (mammary architecture and microenvironment engineering) cultures [32–33]. In MAME cultures, TNBC cells form structures resembling *in vivo* tumors [14] and by using the live-cell proteolysis assay developed by the Sloane laboratory one can visualize, localize and quantify proteolysis in the MAME cultures in real time [34]. The ability to quantify and monitor with time the proteolytic degradation of ECM proteins by living tumor cells is important to designing protease inhibitors that will be efficacious in cancers [34–35]. To the best of our knowledge this is the first time that photoactivation of a caged inhibitor of CTSB has been demonstrated to block proteolysis at the surface of living cancer cells, in this case living breast cancer cells grown in 3D MAME models that recapitulate the *in vivo* microenvironment of human breast tumors [32–33].

## Materials and Methods

### Cathepsin B Activity Assay

CTSB purified from human liver was purchased from Athens Research & Technology (Athens, GA, USA). The enzyme activity was determined from kinetic measurements performed by fluorimetric detection of the hydrolysis product AMC at 37°C for 30 min at one-minute intervals using a Tecan SpectraFluor Plus plate-reader. The excitation and emission wavelengths were 360 and 485 nm, respectively. The fluorescent substrate Z-Arg-Arg-AMC (Bachem, Torrance, CA, USA) was used at a final concentration of 150 μM.

The enzyme was activated for 15 minutes with activator buffer (100 μl) containing 5 mM EDTA, 10 mM DTT (pH 5.2) at 37°C. The sample (1 μl) was added and the reaction mixtures were conducted during 45 min under dark (no irradiation) and light (irradiation at 250 W, 395–750 nm) conditions with a tungsten halogen lamp and H_2_O filter on separate plates. For the experiments under dark conditions, room lights were off and in addition the plate was covered with aluminum foil (light protection); the other plate was exposed to visible light for the same time period. The irradiation wavelength was selected by placing a 395 long-pass filter between the lamp and the sample, along with a 10 cm water cell to absorb infrared light. After photolysis, the reaction was initiated by addition of 200 μl of assay buffer (0.6 mM CaCl_2_, 0.6 mM MgCl_2_, 25 mM piperazin-N-N‘-bis[2-ethanosulfonic acid] (disodium salt)), pH 7.3 containing 150 μM Z-Arg-Arg-AMC substrate solution. Enzyme concentration was 10 nM. The experiments were carried out in triplicate in 96-well flat bottom black plates at pH 6.0. Enzyme activities are expressed as a percentage, with 100% equal to activity in the absence of inhibitor. IC_50_ values were determined by plotting percent activity vs. log (inhibitor concentration).

### Inhibition of Cellular Cathepsin B Activity

TNBC cell lines were originally purchased from American Type Culture Collection (Rockville, MD, USA) and authenticated using the STR PowerPlex 16 system (Promega). Cells were cultured in basal medium supplemented with 10% fetal bovine serum (MDA-MB-231, DMEM; Hs578T, DMEM + 10 μg/ml bovine insulin). To prepare cell lysates, cells were grown on 100 mm dishes to ~80% confluency, washed twice with PBS (phosphate buffered saline), scraped and lysed in SME buffer (250 mM sucrose, 25 mM MES, 1 mM EDTA, pH 6.5, and 0.1% Triton X-100) and then sonicated on ice three times for 10 sec each. CTSB activity was measured as previously described [36]. Briefly, 50 μl of cell lysate was incubated with 300 μl of activator buffer (5 mM EDTA, 10 mM DTT (pH 5.2) at 37°C during 15 min. The sample (1 μl) was added to 100 μl of the activator buffer containing cell lysate and the reaction mixtures were conducted during 45 min under dark (no irradiation) and light (irradiation at 250 W, 395–750 nm) conditions for 45 min with a tungsten halogen lamp and H_2_O filter on separate plates as described above. The experiments were carried out in triplicate (on 96-well flat bottom black plates) at pH 6.0.

### Live-Cell Proteolysis Assay

Proteolytic cleavage of DQ-collagen IV substrate (Invitrogen, Carlsbad, CA, USA) by live TNBC cells was imaged in real time and quantified as previously described [34, 37–38]. Briefly, glass coverslips were coated with 50 μl of 16.4 mg/ml Cultrex^TM^ containing 25 μg/ml DQ-collagen IV and incubated for 15 min at 37°C to solidify. TNBC cells (1.0 x 10^4^) were seeded onto coated glass coverslips and incubated for 60 min. Medium containing 2% Cultrex^TM^ was added to the cells. The sample was added every 48 h. After 4 days of culture, proteolysis of DQ-collagen IV (green fluorescence) was observed in live cells with a Zeiss LSM 780 confocal microscope with a 20X water immersion objective. Where indicated, assays were performed in the presence of CA074 and CA074Me (5 μM each) (Peptides International, Louisville, KY, USA), compound **1** or compound **2**. For experiments with the caged inhibitor, cells were exposed to dark (no irradiation) and light (irradiation at 250 W, 395–750 nm) conditions for 45 min with a tungsten halogen lamp and H_2_O filter on separate plates. After 4 days of culture, optical sections through the entire depth of the 3D structures were acquired on a confocal microscope and, reconstructed in 3D using Volocity software. At the time of imaging cells were stained with CellTracker Orange and nuclei with Hoechst 33342. Volocity software was used to determine cellular boundaries in each confocal slice and generate a “cytoplasmic mask” as well as to count nuclei. The intensity of DQ-collagen IV degradation fragments per cell was quantified in the entire 3D volume. Degradation fragments were segmented into those present inside or surrounding the cells by using image arithmetic as we have previously described in detail [34].

### Cell Viability Assays

Three-dimensional cultures of TNBC cells were established, grown and treated with compound **2** as described above. After 4 days, a cell viability assay was performed to assess the cytotoxic effects of compound 2. The assay has two components: Calcein AM that fluoresces green when cleaved by intracellular esterases thereby labeling live cells and Ethidium Homodimer-1 that fluoresces red when incorporated in the DNA of dead cells. A solution of 4 μM Ethidium Homodimer-1 and 2 μM Calcein AM was prepared in sterile PBS. Cultures were incubated for 30 minutes at 37°C, washed once with warm PBS and replenished with warm MEGM media for live cell imaging. The samples were then imaged at 10X magnification using a Zeiss 510 META confocal microscope. Tiled 16-panel images and z-stacks through the depth of structures were captured. The images were processed to show top views using Volocity software.

### Statistical Analyses

Statistics were performed using the data analysis package within GraphPad Prism 6.0 (GraphPad Sofware, San Diego, CA, USA). Unless otherwise stated, tests comparing two means are Student’s t-test, with equal variance assumed.

### Synthesis of Δ- and Λ-cis [Ru(bpy)_2_(1)_2_](BF_4_)_2_ (2)

In the glove box, a sealable tube was charged with *cis*-Ru(bpy)_2_Cl_2_ (0.062 mmol 30 mg), AgBF_4_ (0.24 mmol 47.9 mg) and (S)-3,4-dichloro-N-(1-((cyanomethyl)amino)-1-oxo-3-(m-tolyl)propan-2-yl)benzamide [39] (**2**) (0.37 mmol 145.3 mg) and freshly distilled EtOH (15 mL). The resulting solution was wrapped in aluminum foil and refluxed for 6 h during which time it turned from dark violet to bright orange. After cooling the crude solution to RT, it was placed in the freezer at –20°C for 16 h. The precipitated silver salts were filtered off using celite and the filter cake was washed with cold EtOH (190 proof). The solvents were removed under reduced pressure and the crude mixture was analyzed by ^1^H NMR spectroscopy. The resulting yellow solid was dissolved in acetone (2 mL) and layered with Et_2_O (10 mL) and placed in the freezer at –20°C for 16 h. The solution was decanted, and the solid was suspended in acetone (5 mL), then treated with Ag-scavenging silica gel (QuadraSil® MP, 20 mg). After 15 min, the suspension was filtered and the solution was concentrated. The resulting solid was stirred with EtOAc (15 mL) for 4 h. The orange solid was centrifuged and the resulting filtered cake was washed with Et_2_O (3 ×15 mL) and dried under reduced pressure to give the title compound as a yellow solid in analytically pure form as a hydrate salt (47 mg, 55%). mp = 178 °C (decomp); ^1^H NMR (400MHz C_3_D_6_O) δ 9.51 (m, 2H), 8.79–8.75 (m, 2H), 8.66 (d, 2H, J = 8.4 Hz), 8.37–8.20 (m, 6H), 8.08 (t, 2H, J = 7.2 Hz), 7.89–7.70 (m, 8H), 7.62–7.61(m, 2H), 7.41–7.40 (m, 2H), 7.12–7.06 (m, 6H), 6.98 (t, 2H, J = 7.2 Hz), 4.80–4.75 (m, 2H), 4.58–4.55 (m, 1H), 4.47–4.44 (m, 3H), 3.21–3.18 (m, 2H), 3.07–3.00 (m, 2H), 2.22 (s, 3H), 2.19 (s, 3H); IR (KBr) ν_max_ (cm^-1^) 3618, 3567, 3374, 3083, 2921, 2278, 1664, 1606, 1591, 1526, 1467, 1447, 1426, 1379, 1340, 1312, 1276, 1242, 1161, 1125, 1009, 1062, 1032, 895, 834, 767, 731, 701, 675. ESMS calcd for C_58_H_50_BCl_4_F_4_N_10_O_4_Ru (M^+1^ **1**-BF_4_) 1281.18, found 1281.23; UV-Vis λ_max_ = 420 nm (ε = 10,360 M^-1^cm^-1^); Anal. Calcd for C_58_H_60_B_2_Cl_4_F_8_N_10_O_9_Ru (**1·**5H_2_O): C, 47.79; H, 4.15; N, 9.61. Found: C, 47.45; H, 4.20; N, 9.62, water content was confirmed by ^1^H NMR spectroscopy.

### Photoinduced Ligand Exchange

Electronic absorption spectra were measured with a Hewlett-Packard 8453 diode array spectrophotometer. The ligand exchange quantum yield experiments were performed with a 150 W Xe arc lamp (USHIO) in a Milliarc lamp housing unit with an LPS-220 power supply and an LPS-221 igniter (PTI). A bandpass filter (Thorlabs) and long-pass filter (CVI Melles Griot) were used to select the appropriate irradiation wavelengths.

The photolysis experiments were performed in H_2_O with 2% acetone and in an acetone solution containing 0.25 M tetrabutylammonium, and the chloride (TBACl) in a 1 × 1 cm quartz cuvette, and the irradiation wavelengths were selected with a 395 nm long-pass filter. The photon flux of the arc lamp with a 335 nm long-pass filter and a 400 nm bandpass filter was determined to be 4.30 ± 0.25 × 10^−8^ mol photons/min by ferrioxalate actinometry as previously described in detail [40]. The quantum yields (Φ) for ligand dissociation of **2** were determined in each of the solvent systems in a 1 × 1 cm quartz cuvette. The decrease in the MLCT absorption maximum of the reactant with ε = 10,360 M^–1^cm^–1^ at 412 nm as a function of time was monitored at early irradiation times to calculate the rate of moles of reactant converted to intermediate, I. The increase of the MLCT absorption maximum of the product at 490 nm (ε = 9,300 M^–1^cm^–1^) [41] was monitored at irradiation times after the formation of the intermediate to determine the rate of moles of product P formed. These rates along with the photon flux were used to calculate the quantum yields for the first and second steps, Φ_R→I_ and Φ_I→P_, respectively.

## Results and Discussion

### Evaluation against purified CTSB and breast tumor cell lysates

A dipeptidyl nitrile CTSB inhibitor (**1)** and its caged version [Ru(bpy)_2_(**1**)_2_](BF_4_)_2_ (**2**) were evaluated against CTSB purified from human liver and IC_50_ values were determined. Compound **1** showed an IC_50_ value of 0.33 μM, similar to data previously reported for inhibition of recombinant human CTSB expressed in baculovirus [39]. The IC_50_ values for the caged variant **2** under light (λ_irr_ > 395 nm) vs. dark conditions were 0.28 and 3.4 μM, respectively, corresponding to a dark/light IC_50_ ratio of 12 (Table 1). As expected, caged **2** became more active under irradiation than in the dark, consistent with the dissociation of the dipeptidyl nitrile molecules from the ruthenium moiety used as a photocage. The potency of the uncaged **1** was very similar to that of irradiated caged **2**, i.e., 0.33 and 0.28 μM, respectively. The strong similarity between the IC_50_ values for the uncaged and caged inhibitors could be related to the lower efficiency for the release of the second molecule of inhibitor from **2**, as shown previously for caged inhibitors of cathepsin K [30–31].

**Table 1:** IC_50_ values^a^ (μM) for compounds 1 and 2 and dark/light ratio (with and without irradiation) against human CTSB and human breast cancer cell lysates.

	IC_50_ (μM)		
	1	2 (dark)	2 (light)
CTSB	0.33	3.4	0.28
MDA-MB-231	1.54	125.8	1.58
Hs578T	0.91	11.2	0.89

^a^Inhibitory activities were determined with the fluorogenic substrate Z-Arg-Arg-AMC.

Reactions were conducted in the dark (no irradiation) and light (λ_irr_ = 395–750 nm) for 45 min with a 250 W tungsten halogen lamp and H_2_O filter. The standard deviations were within 40% of the IC_50_ values and the curves were plotted against log [inhibitor] with 100% activity set equal to the control reaction in the absence of inhibitor. Assay conditions: activator buffer containing 5 mM EDTA, 10 mM DTT, pH 5.2 and assay buffer containing 0.6 mM CaCl_2_, 0.6 mM MgCl_2_, 25 mM piperazin-N-N‘-bis[2-ethanosulfonic acid (disodium salt)], pH 7.3. DMSO was used as a negative control. Substrate concentration was 150 μM and CTSB concentration was 10 nM. Data shown are from 3 independent experiments.

We evaluated the ability of compounds **1** and **2** to inhibit proteolytic activity in lysates of two TNBC cell lines grown in monolayer cultures on plastic dishes: MDA-MB-231 and Hs578T. The substrate used was Z-Arg-Arg-AMC, a substrate that under the conditions of our assay is highly selective for CTSB [14, 36]. In MDA-MB-231 cell lysates the photocaged compound **2** was significantly more potent when irradiated than it was in the dark with a dark/light ratio of 79.6 (Table 1). Similar results were obtained with Hs578T cell lysates although the dark/light ratio was lower, i.e., 12.6. The higher dark/light ratio for MDA-MB-231 may be due to differences in CTSB activity in the two cell lines (data not shown); however, the IC_50_ values for inhibitor **1** and light-activated inhibitor **2** were comparable. The MDA-MB-231 cell line also has been shown to exhibit 10-fold more sensitivity to a statin inhibitor than does the Hs578t cell line [42]. Our analyses using lysates of two TNBC cell lines that are classified as mesenchymal stem-like [43] and purified human CTSB confirmed the efficiency of light activation of the photocaged inhibitor.

#### 3D MAME Cell Culture Assays for Breast Cancer Cell Lines

**Fig 1:**
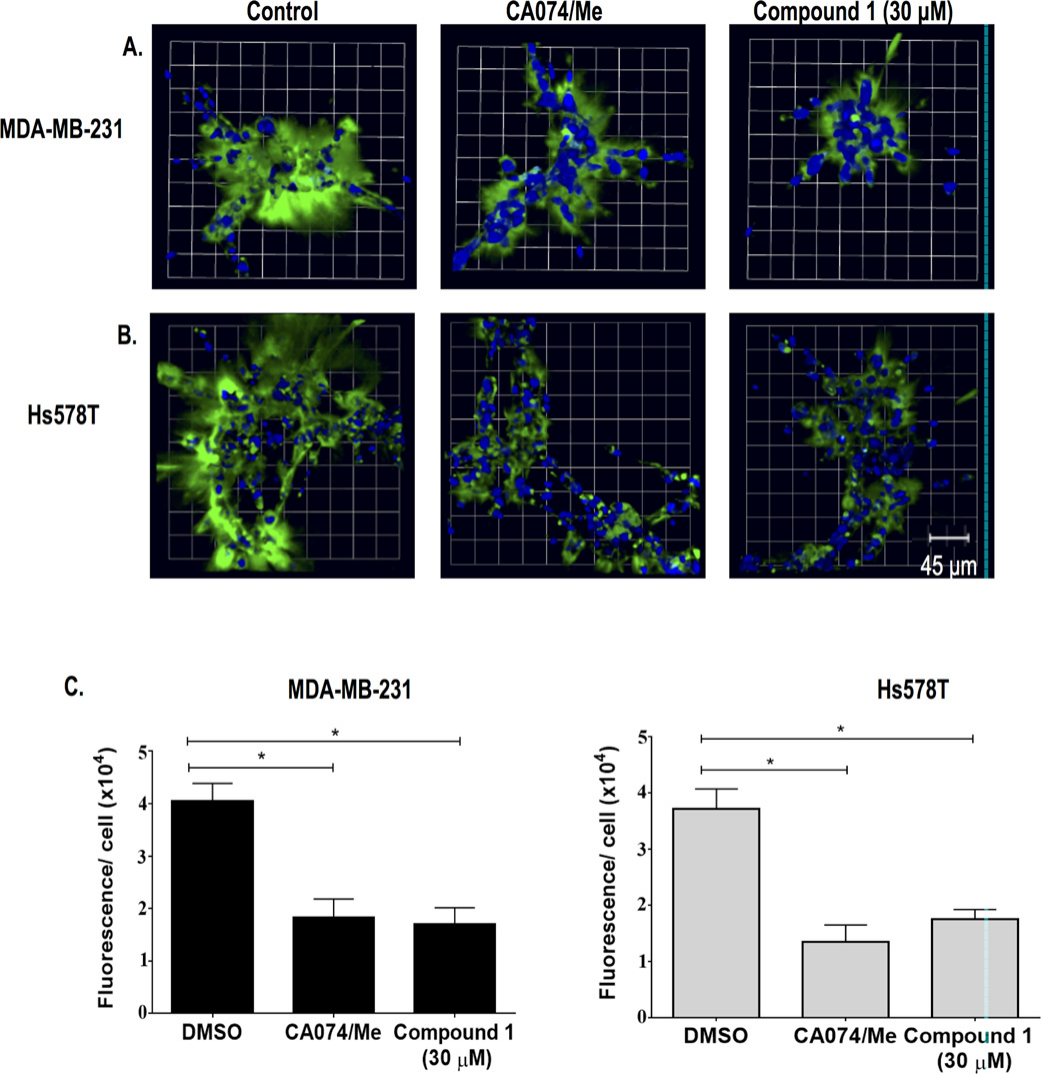
Uncaged inhibitor 1 reduces degradation of DQ-collagen IV by 3D MAME cultures of breast carcinoma cells. (A) Top view of representative 3D reconstruction of 16 contiguous fields of MDA-MB-231 breast carcinoma structures (nuclei, blue) and associated degradation fragments of DQ-collagen IV (green) at 4 days of culture. Panels from left to right are DMSO control and cysteine protease inhibitors (middle: 5 μM each of CA074 + CA074Me; right: uncaged inhibitor 1). (B) Hs578T breast carcinoma structures (nuclei, blue) and associated degradation fragments of DQ-collagen IV (green) at 4 days of culture. See A for further details. (C) Quantification of degraded DQ-collagen IV per cell in MDA-MB-231 (left) and Hs578T (right) structures exposed to DMSO (negative control), CA074/CA074Me (5 μM each; positive control) and uncaged inhibitor **1**. Data shown are from 3 independent experiments (48 fields); * ≤ 0.05; mean ± SD.

We tested the ability of the inhibitors to reduce CTSB activity in intact human TNBC cells grown in 3D MAME cultures. Studies using 3D human pathomimetic models have demonstrated the importance of evaluating molecular and cellular function in a model that mimics disease progression by approximating organ structure. Such models encompass cell–cell and cell–matrix interactions [6, 35]. According to Schmeichel and Bissell, 3D models have the potential when used for drug screening to identify and validate molecules that will sustain efficacy in clinical trials [35]. We employed a live-cell proteolysis assay developed in the Sloane laboratory [34] in which dye quenched fluorescent proteins are the proteolytic substrates (Fig 1). The fluorescent signal in this gain-of-function assay allows us to localize the site of degradation and is proportional to the proteolytic activity [34]. The protein substrate can be cleaved by many proteases allowing the analysis of proteolytic networks rather than a single protease or protease class as is the case with assays employing selective synthetic small molecule substrates. Here we grew MDA-MB-231 and Hs578T TNBC breast cancer cells in 3D MAME cultures containing DQ-collagen IV [14] and assessed the formation of degradation products of DQ-collagen IV (green fluorescence) in real-time. We found that the uncaged inhibitor **1** reduced degradation of DQ-collagen IV (Fig 1A and 1B). Quantification of the intensity of fluorescent degradation products per cell in the entire 3D volume of TNBC structures revealed significantly less degradation of DQ-collagen IV in the presence of uncaged inhibitor **1** (Fig 1C). To compare the efficacy of uncaged inhibitor 1 to other inhibitors that are selective for CTSB, we used a mixture of CA074 and CA074Me [44], which are non-cell permeable and cell permeable, respectively, and thus should inhibit both pericellular and intracellular CTSB. With this mixture, we observed a reduction in degradation of DQ-collagen IV comparable to that observed with uncaged inhibitor 1 (Fig 1). CA074Me, but not CA074, inhibits cathepsin L [44], another cysteine cathepsin considered a target for treatment of cancer [45].

**Fig 2:**
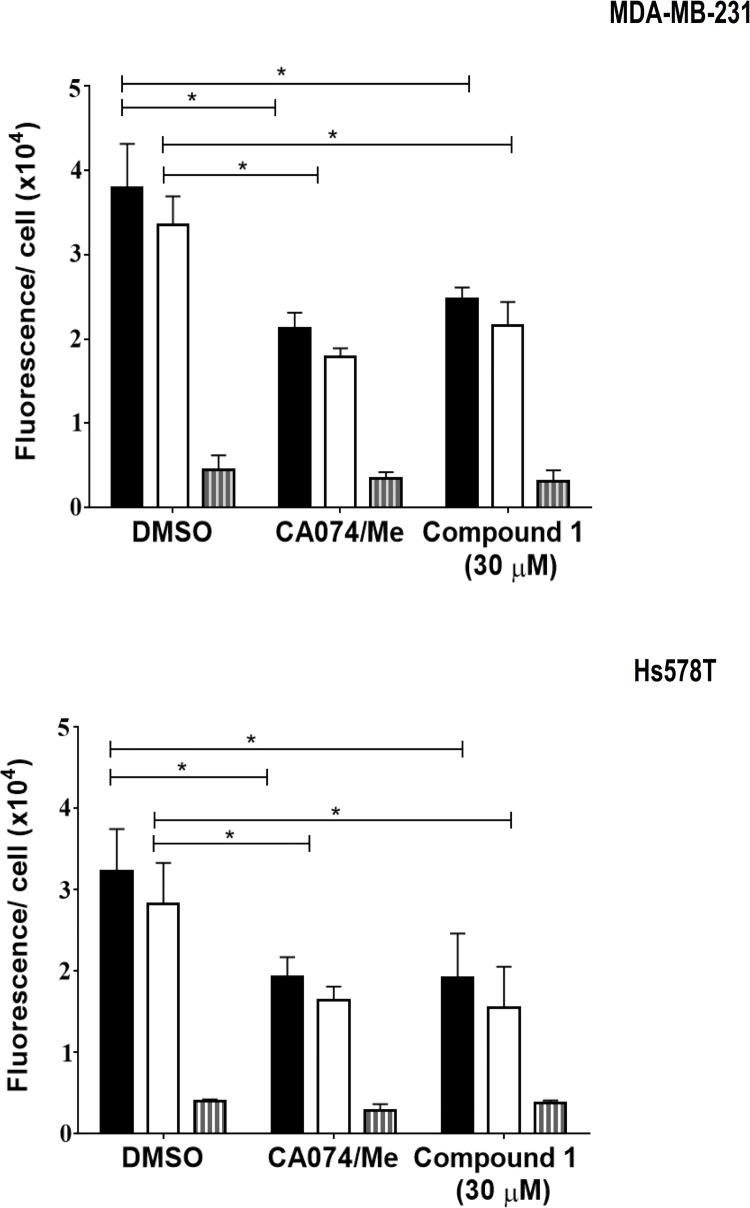
Uncaged inhibitor 1 reduces total and pericellular degradation, but not intracellular degradation of DQ-collagen IV by 3D MAME cultures of breast carcinoma cells. Quantification of degraded collagen IV in entire 3D volume of MDA-MB-231 and Hs578T structures at 4 days of culture: total degraded collagen IV, black bars; pericellular degraded collagen IV, open bars; and intracellular degraded collagen IV, gray bars. DMSO (negative control), CA074/CA074Me (5 μM each; positive control) and uncaged inhibitor 1. Data shown are from 3 independent experiments (48 fields); * p < 0.05; mean ± SD.

In cancers, the cellular localization of CTSB is often altered such that the enzyme is distributed at the cell surface or secreted [46–48]. CTSB has been localized to a number of cell surface structures associated with proteolysis and invasion, e.g., caveolae [49,50], podosomes [51] and invadopodia [52,53]. Increases in CTSB activity at the cell surface occur in response to peritumoral acidosis and have been shown to mediate increases in degradation of extracellular matrix proteins [14] and invasion [51,54]. Thus, when assessing the efficacy of CTSB inhibitors it is important to determine the site at which a protease is inhibited [14–15]. This is an advantage of the live-cell proteolysis assay used here, i.e., degradation fragments can be localized to pericellular or intracellular compartments [14, 34, 50]. This allowed us to determine potential differences in the ability of the uncaged inhibitor **1** to reduce total, pericellular or intracellular proteolysis by the MDA-MB-231 and Hs578T 3D MAME cultures. We observed significant reductions in total proteolysis and pericellular proteolysis, but not in intracellular proteolysis, in the presence of the uncaged inhibitor **1** and comparable results with the mixture of highly selective CTSB inhibitors (Fig 2). These results are consistent with the uncaged inhibitor **1** acting through inhibition of pericellular CTSB.

**Fig 3:**
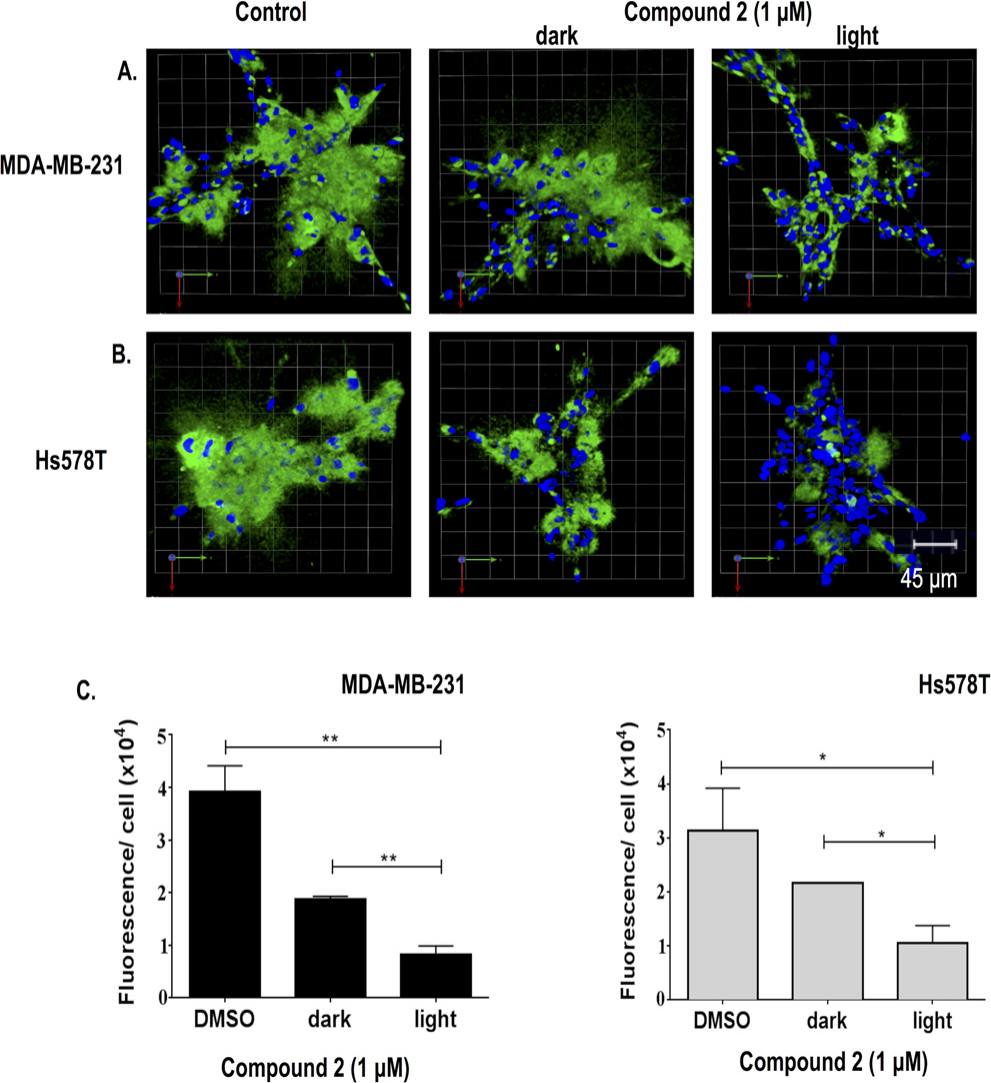
Light activation of caged inhibitor 2 reduces degradation of DQ-collagen IV by 3D MAME cultures of breast carcinoma cells. (A) Top view of representative 3D reconstruction of 16 contiguous fields of MDA-MB-231 breast carcinoma structures (nuclei, blue) and associated degradation fragments of DQ-collagen IV (green) at 4 days of culture. Panels from left to right are DMSO control, dark-exposed caged inhibitor **2** and light-exposed caged inhibitor **2**. (B) Hs578T breast carcinoma structures (nuclei, blue) and associated degradation fragments of DQ-collagen IV (green) at 4 days of culture. See A for further details. (C) Quantification of degraded DQ-collagen IV per cell in MDA-MB-231 (left) and Hs578T (right) structures incubated with DMSO (negative control), dark-exposed caged inhibitor **2** or light-exposed caged inhibitor **2**. Data shown are from 3 independent experiments (48 fields); *p ≤ 0.05; **p ≤ 0.005; mean ± SD.

In order to prove the efficacy of the caging strategy, the caged compound **2**, a light-activated enzyme inhibitor, was evaluated in live-cell proteolysis assays in the dark and the light (Fig 3). At a concentration of 1 μM, photoactivated compound **2** significantly reduced the degradation of DQ-collagen IV (green fluorescence) in 3D MAME cultures of TNBC cells exposed to visible light. Initially the assay was carried out with higher concentrations of compound **2**; however, significant inhibition of proteolysis was observed in the dark (data not shown). Reducing the concentration of compound **2** resulted in an increase in the dark/light ratio. Inhibition by **2** in the dark likely indicates that inhibitor **1** is partially released over the timescale of the experiment (4 days), consistent with the shorter half-life of **2** (t_1/2_ ~ 10 days) as compared to other Ru^II^(bpy)_2_ caged inhibitors we have examined thus far [30–31]. Therefore, further optimization of the caging group and/or inhibitor may be necessary to achieve higher dark to light ratios in these assays. Nonetheless, the live-cell proteolysis assay demonstrated that the caged inhibitor was more potent than the uncaged inhibitor and can be used at a much lower concentration, an advantage for use of the caged inhibitor *in vivo*.

**Fig 4:**
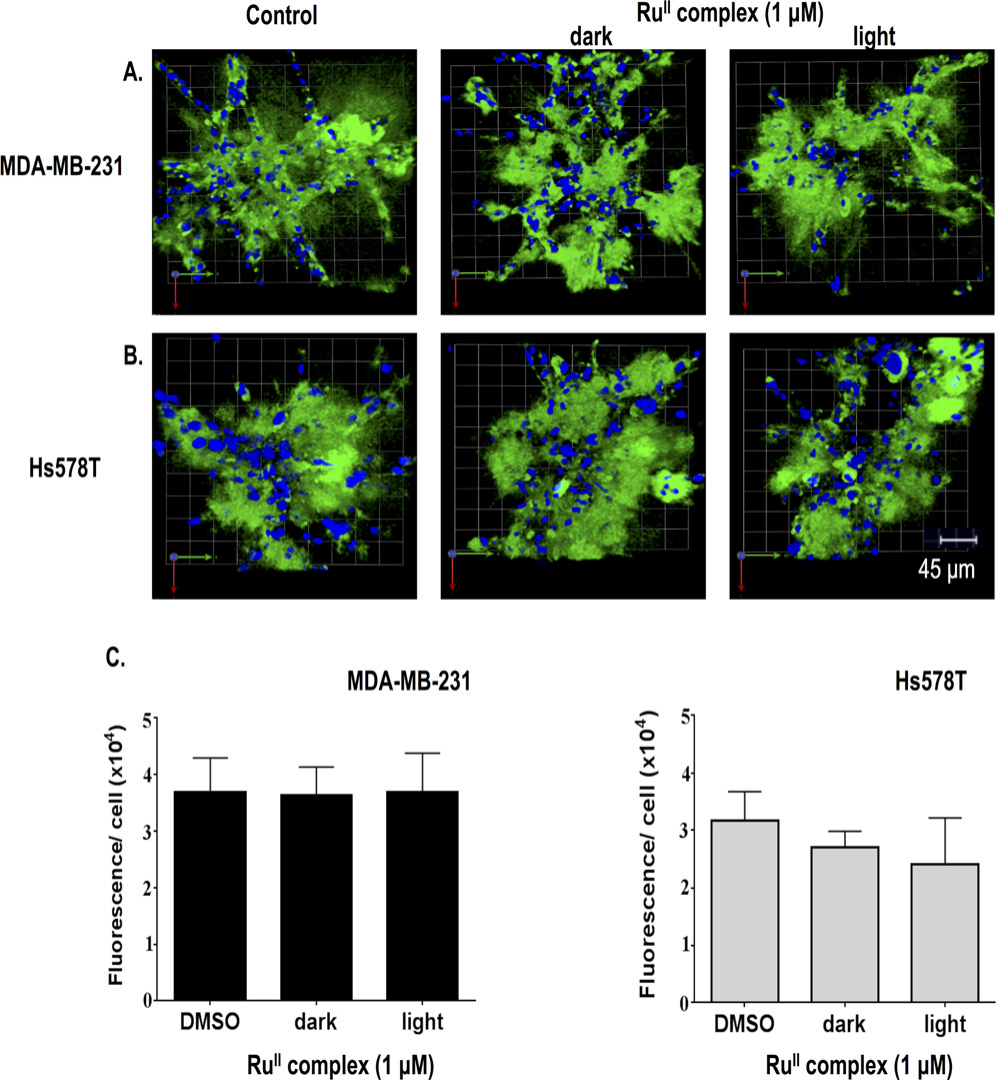
The ruthenium complex *cis*-[Ru(bpy)_2_(MeCN)_2_](PF_6_)_2_ (3) used for caging of inhibitor 2 does not affect degradation of DQ-collagen IV by 3D MAME cultures of breast carcinoma cells. (A) Top view of representative 3D reconstruction of 16 contiguous fields of MDA-MB-231 breast carcinoma structures (nuclei, blue) and associated degradation fragments of DQ-collagen IV (green) at 4 days of culture. Panels from left to right are DMSO control, dark-exposed ruthenium complex and light-exposed ruthenium complex. (B) Hs578T breast carcinoma structures (nuclei, blue) and associated degradation fragments of DQ-collagen IV (green) at 4 days of culture. See A for further details. (C) Quantification of degraded DQ-collagen IV per cell in MDA-MB-231 (left) and Hs578T (right) structures incubated with DMSO (negative control), dark-exposed ruthenium complex or light-exposed ruthenium complex. Data shown are from 3 independent experiments (48 fields); mean ± SD.

Having established that caged inhibitor **2** was able to block proteolysis in a light activated fashion, experiments with only the ruthenium caging group, i.e., compound **3**, were carried out in the absence and presence of light to determine the ability of compound **3** to reduce proteolysis. Control experiments on 3D MAME cultures with a 1 μM concentration of inhibitor under light vs. dark conditions proved that *cis*-[Ru(bpy)_2_(MeCN)_2_](PF_6_)_2_ (**3**) did not inhibit proteolysis (Fig 4A and 4B) as no differences in fluorescence were observed in the images. Furthermore, quantification established that the amount of green fluorescence per cell was the same as in the vehicle control (1% DMSO) (Fig 4C). We did not observe any toxicity of compound **2** as determined by cell viability assays of MDA-MB-231 and Hs578T 3D MAME cultures in the dark or the light (S1 Fig). Our results are consistent with data from the literature demonstrating that the caging fragment Ru^II^(bpy)_2_ does not exhibit side effects or toxicity [24, 31].

### Synthesis and characterization of dipeptidyl nitrile inhibitor 1 and 2

**Fig 5:**
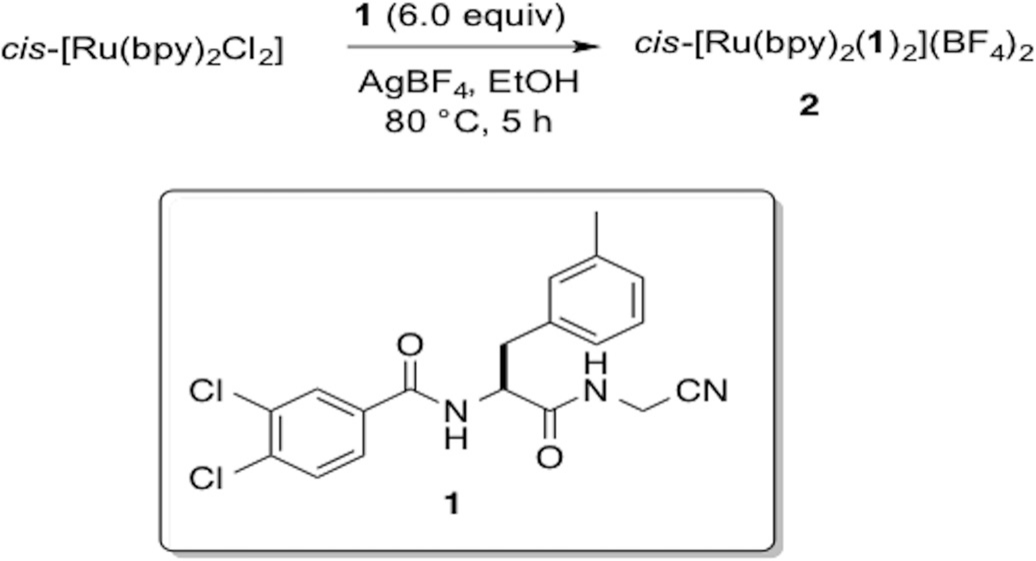
Synthesis of the ruthenium-caged, nitrile-based inhibitor 2.

Dipeptidyl nitrile **1** was chosen for caging in this study, because it was identified previously as a potent, selective and reversible inhibitor of recombinant human CTSB expressed in baculovirus (Fig 5) [39]. Caging of inhibitor **1** was accomplished by treating **1** (6.0 equiv) with *cis*-[Ru(bpy)_2_Cl_2_] (1.0 equiv), and AgBF_4_ (4.0 equiv) in EtOH at 80°C, resulting in a color change from violet to orange. Cooling of the crude reaction mixture to –20°C, followed by filtration and concentration gave crude **2**, which was obtained as the major ruthenium-based product, as judged by ^1^H NMR spectroscopic analysis. Compound **2** was purified further by multiple cycles of stirring with diethyl ether to remove unreacted **1**, precipitation from a solution of acetone by diethyl ether vapor diffusion, treatment with QuadraSil® MP, a thiol-based Ag-scavenging silica gel, and washing with EtOAc and Et_2_O, which provided **2** as a hydrate salt in an analytically pure form.

Caged inhibitor **2** was characterized by ^1^H NMR, IR and UV-Vis spectroscopies, mass spectrometry and elemental analysis. ^1^H NMR spectroscopic data were consistent with **2** being isolated as a 1:1 mixture of Δ- and Λ stereoisomers, as shown previously [30–31]. This was expected because *cis*-[Ru(bpy)_2_Cl_2_] was used as a racemic mixture of Δ- and Λ stereoisomers. The IR spectrum of **2** shows a ν_CN_ stretch at 2278 cm^-1^, diagnostic of nitrile binding to Ru(II). The electrospray ionization mass spectrum of **2** shows prominent peaks at m/z = 1281.2, along with a suitable isotopic pattern, consistent with a monocation of the formula [Ru(bpy)_2_(**1**)_2_](BF_4_)^+^. The electronic absorption spectrum of **2** is highly consistent with other dications of the general formula *cis*-[Ru(bpy)_2_(RCN)_2_]^2+^, where RCN is MeCN,^130^ 5-cyanouracil,^124^ and protease inhibitors structurally related to **1** [17–18]. Data for **2** in water containing 2% acetone show a maximum at 420 nm (ε = 10,360 M^-1^cm^-1^) assigned to a singlet metal-to-ligand charge transfer (^1^MLCT) transition. The half-life for **2** in the dark was determined spectrophotometrically using the rate constant for decomposition, obtained from the slope of a ln A vs. t graph in DMSO (k_obs_ = 1.6 ± 0.1 × 10^−7^ s^-1^) and phosphate buffered saline (PBS, pH 6.5, k_obs_ = 1.2 ± 0.1 × 10^−6^ s^-1^) as described previously [30–31], to be ~70 and ~10 days, respectively at 293 ± 2 K.

**Fig 6:**
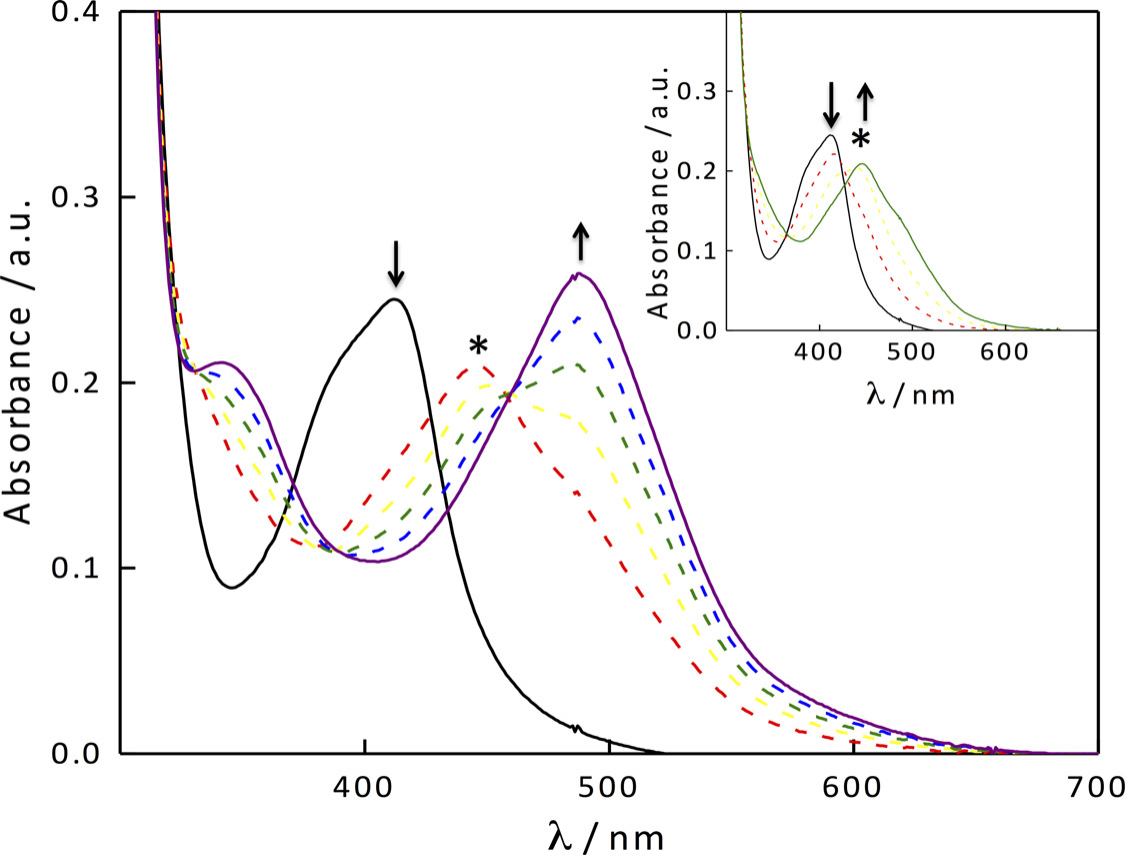
Changes to the electronic absorption of compound 2 (25 μM) in a 2% acetone aqueous solution at irradiation times, t_irr_, of 0, 3, 5, 7, 10 and 15 min (λ_irr_ ≥ 395 nm); the * denotes the mono-aqua intermediate. Inset: t_irr_ = 0.0, 0.5, 1.5, and 3.0 min.

The photochemical reactivity of ruthenium-caged complex **2** was evaluated by monitoring the changes to the electronic absorption spectrum of the complex as a function of irradiation time (λ_irr_ ≥ 395 nm) in a 2% acetone aqueous solution (Fig 6). The changes to the absorption spectrum of **2** are consistent with those of the related [Ru(bpy)_2_(RCN)_2_]^2+^ complexes [29–31,51]. A decrease of the reactant ^1^MLCT transition at 412 nm and evolution of a new transition at 446 nm at early irradiation times (up to 3 min) can be assigned as arising from the photoinduced substitution of one nitrile ligand, **1**, by a solvent H_2_O molecule to form a mono-aqua intermediate, [Ru(bpy)_2_(**1**)(OH_2_)]^2+^ (I). This process exhibits an isosbestic point at 427 nm indicative that consumption of the reactant is directly correlated to intermediate formation. Further irradiation up to 15 min results in formation of the bis-aqua product, [Ru(bpy)_2_(OH_2_)_2_]^2+^ (P) via substitution of the remaining **1** ligand with H_2_O. This process is indicated by a decrease of the peak of the intermediate at 446 nm, the appearance of a new transition at 487 nm, and isosbestic points at 383 and 460 nm. The quantum yield of ligand exchange for the first step, reactant R to intermediate I (Φ_R→I_) was determined to be 0.026(4), while the second step, from intermediate I to product P (Φ_I→P_) was measured to be 0.0045(9) in water (λ_irr_ = 400 nm). The relatively low quantum yield values can be attributed to the low solubility of **1** in H_2_O, such that following photoinduced nitrile dissociation, the ligand does not exit the solvent cage efficiently and recombines with the pentacoordinate ruthenium fragment to regenerate the starting material. Because **1** is more soluble in acetone, the photosubstitution reaction was repeated in an acetone solution containing 0.25 M tetrabutylammonium chloride (TBACl). In this system, irradiation of **2** forms the intermediate [Ru(bpy)_2_(**1**)Cl]^+^ followed by a second ligand substitution to form [Ru(bpy)_2_Cl_2_]. The quantum yields in this solvent are greatly enhanced, with Φ_R→I_ and Φ_I→P_ of 0.056(9) and 0.032, respectively. This experiment clearly shows that the ligand exchange quantum yields of caged compounds, such as **2**, can be enhanced by increasing the water solubility of the nitrile inhibitor.

## Conclusion

CTSB, one of the most abundant cysteine cathepsins, is upregulated in breast and other cancers and considered to be an attractive molecular target for cancer therapy. Here we report the successful application of a photoactivated CTSB inhibitor (**2**) to block proteolysis in live-cell assays of 3D MAME TNBC models, which offer a unique system for dynamic imaging of proteolysis. These models mimic the *in vivo* microenvironment of human breast cancers and allow us to visualize, localize and quantify collagen IV degradation in real time. Our data indicate that compound **1** is as effective as the highly selective CTSB inhibitor CA074 at blocking pericellular proteolysis. Positive results were also obtained in activity assays of purified CTSB and lysates of the 3D MAME TNBC models. These data also provide evidence that the ruthenium caging strategy can be used to garner control over inhibition with light. Although further optimization of caged inhibitors will be necessary to afford compounds with high stability and better dark to light ratios, our complexes were not toxic and the caging group did not inhibit proteolysis. Importantly, this example serves as a prototype for development of biochemical tools for further analysis of proteases in cancer and clinically relevant, photoactivated inhibitors of CTSB with red-shifted absorbance to facilitate tissue penetration by low energy light for *in vivo* applications. Also, by increasing our understanding of the role played by ECM components in the tumor microenvironment and their potential impact on protease inhibition, we can provide more reliable data for further assays searching for CTSB inhibitors in cancer tumors.

## Supporting Information

S1 Fig Light activation of caged inhibitor 2 did not affect the viability of 3D MAME cultures of breast carcinoma cells, as assessed by Live/Dead assays. (A) Top view of representative 3D reconstruction of 16 contiguous fields of MDA-MB-231 breast carcinoma structures following an assay for cell viability at 4 days of culture. Panels from left to right are DMSO control, dark-exposed caged inhibitor **2** and light-exposed caged inhibitor **2**. Viable cells fluoresce green and dead cells fluoresce red. (B) Hs578T breast carcinoma structures following an assay for cell viability at 4 days of culture. See A for further details.
(TIFF)

S2 Fig ^1^H NMR of complex 2 (C_3_D_6_O). (TIFF)

S3 Fig COSY spectrum of complex 2 (C_3_D_6_O). (TIFF)

S4 Fig IR spectrum (KBr) of complex 2. (TIFF)
