# Supplementary figures and images for: Imaging Sites of Inhibition of Proteolysis in Pathomimetic Human Breast Cancer Cultures by Light-Activated Ruthenium Compound
Source: PLoS One. 2015 Nov 12;10(11):e0142527. doi: 10.1371/journal.pone.0142527 (PMC4643019; doi:10.1371/journal.pone.0142527)

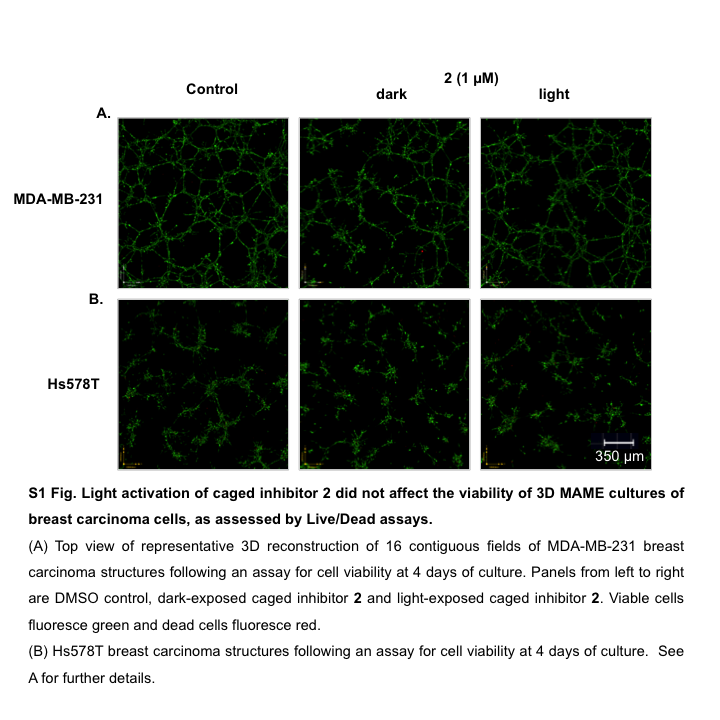

Supplement: S1 Fig — (A) Top view of representative 3D reconstruction of 16 contiguous fields of MDA-MB-231 breast carcinoma structures following an assay for cell viability at 4 days of culture. Panels from left to right are DMSO control, dark-exposed caged inhibitor 2 and light-exposed caged inhibitor 2. Viable cells fluoresce green and dead cells fluoresce red. (B) Hs578T breast carcinoma structures following an assay for cell viability at 4 days of culture. See A for further details. (TIFF) [file pone.0142527.s001.tiff]

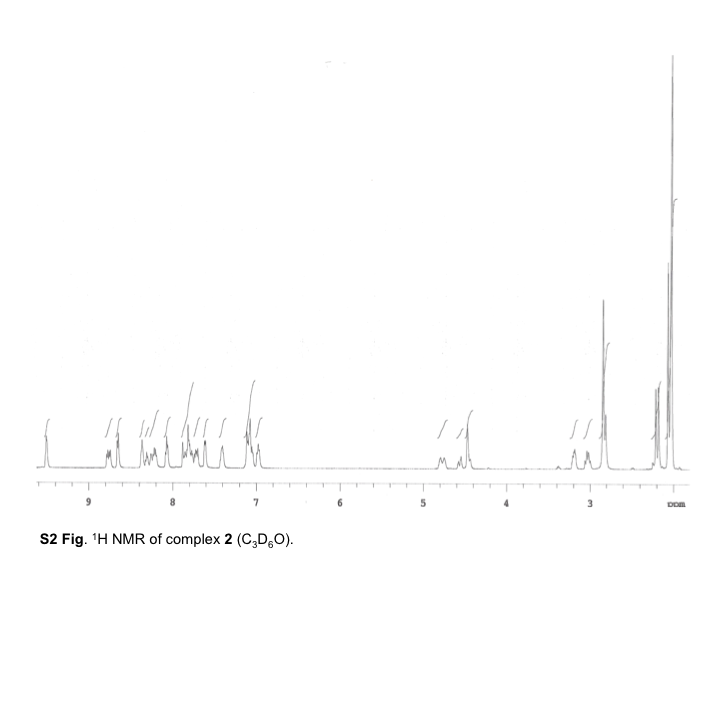

Supplement: S2 Fig — (TIFF) [file pone.0142527.s002.tiff]

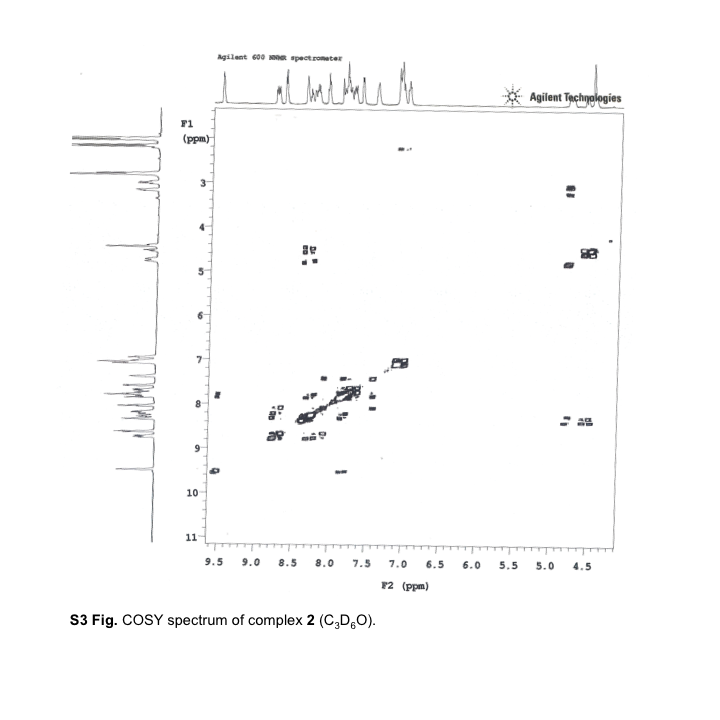

Supplement: S3 Fig — (TIFF) [file pone.0142527.s003.tiff]

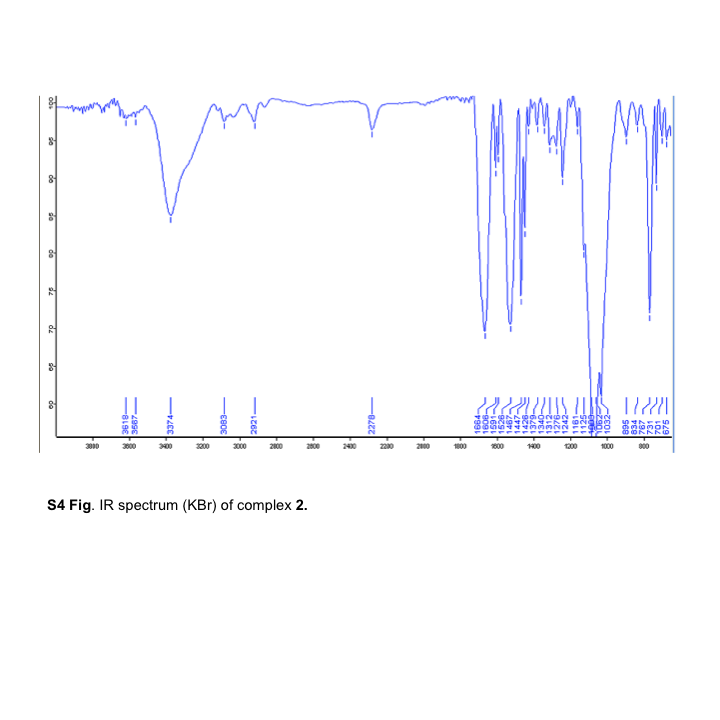

Supplement: S4 Fig — (TIFF) [file pone.0142527.s004.tiff]
